# A highly sensitive liquid chromatography electrospray ionization mass spectrometry method for quantification of TMA, TMAO and creatinine in mouse urine

**DOI:** 10.1016/j.mex.2017.09.004

**Published:** 2017-09-28

**Authors:** Sunil Veeravalli, Kersti Karu, Ian R. Phillips, Elizabeth A. Shephard

**Affiliations:** a Institute of Structural and Molecular Biology, University College London, London, UK; b Mass Spectrometry Facility, Department of Chemistry, University College London, London, UK; c School of Biological and Chemical Sciences, Queen Mary University of London, London, UK

**Keywords:** Quantification of TMA, TMAO and, Creatinine using LC-ESI-MS, FMO3, Mouse urine, Trimethylamine, Trimethylamine N-oxide, Liquid Chromatography, Electrospray ionization, Mass spectrometry

## Abstract

Our method describes the quantification in mouse urine of trimethylamine (TMA), trimethylamine *N*-oxide (TMAO) and creatinine. The method combines derivatization of TMA, with ethyl bromoacetate, and LC chromatographic separation on an ACE C_18_ column. The effluent was continuously electrosprayed into the linear ion trap mass spectrometer (LTQ), which operated in selective ion monitoring (SIM) modes set for targeted analytes and their internal standards (IS). All validation parameters were within acceptable ranges of analytical method validation guidelines. Intra- and inter-day assay precision and accuracy coefficients of variation were <3.1%, and recoveries for TMA and TMAO were 97–104%. The method developed uses a two-step procedure. Firstly, TMA and TMAO are analyzed without a purification step using a 5-min gradient cap-LC- SIMs analysis, then creatinine is analyzed using the same experimental conditions. The method is robust, highly sensitive, reproducible and has the high-throughput capability of detecting TMA, TMAO and creatinine at on-column concentrations as low as 28 pg/mL, 115 pg/mL and 1 ng/mL, respectively. The method is suitable for analysis of TMA, TMAO and creatinine in both male and female mouse urine.

The key benefits of the method are:

• The small sample volume of urine required, which overcomes the difficulties of collecting sufficient volumes of urine at defined times.
• No sample pre-treatment is necessary.
• The quantification of TMA, TMAO and creatinine using the same cap-LC-MS method.

## Graphical abstract

**Figure:**
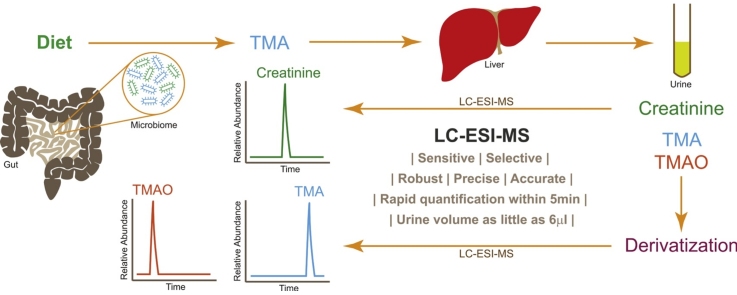

## Method details

### Background

In mammals, trimethylamine (TMA) is liberated by the action of gut bacteria on a number of dietary components [1], [2], [3], [4]. The released TMA is rapidly absorbed and converted in the liver to trimethylamine *N-*oxide (TMAO) in a reaction catalysed by the enzyme FMO3 [5]. TMA and TMAO are excreted in urine [6]. The conversion of TMA to TMAO is impaired in individuals with the rare disorder primary trimethylaminuria [7]. Consequently, such individuals excrete large amounts of TMA in urine. The condition is caused by mutations in the *FMO3* gene [5], [8]. Certain common polymorphic variants of *FMO3*, when present in the homozygous state, can result in mild or transient trimethylaminuria [9]. Changes in the amounts of TMA or TMAO in biological samples have also been reported for disorders of the liver [10] and kidney [11], [12], [13] and for pancreatic cancer [14]. In addition, increased production of TMAO has also been implicated as a risk factor in cardiovascular disease [15]. Measurements of TMA and TMAO in bodily fluids such as plasma or urine are therefore being used increasingly to monitor changes in disease state and/or as an indication of altered gut microbial action.

Several methods have been reported for the determination of TMA and TMAO in human urine, most of which have been developed for the diagnosis of trimethylaminuria. These include gas chromatography (GC) [16], [17], high-performance liquid chromatography (HPLC) [18], proton nuclear magnetic resonance spectroscopy (1H-NMR) [19], [20], [21], fast atom bombardment mass spectrometry (FAB-MS) [22], matrix-assisted laser desorption/ionization with time-of-flight MS (MALDI-TOF-MS) [23] and stable isotope dilution (SID)-LC-MRM-MS [24].

The mouse is often used as a model organism for basic and preclinical research. Collection of urine for metabolite analyses has the advantage of being non-invasive. However, urine cannot be collected ‘on demand’ from an experimental animal. Therefore, we optimised a method for the quantification of TMA and TMAO in mouse urine when sample size is limited and time of collection is important. Johnson [25] developed a flow injection ESI-MS/MS method in which TMA is derivatized with ethyl bromoacetate to form betaine bromide, which improves ionization efficiency using ESI conditions. This method does not include a chromatographic separation step, the absence of which could lead to a suppression of analyte ionization under ESI conditions due to matrix effects. Matrix effects could pose a problem when analyzing male mouse urine, because male mice excrete large amounts of protein in their urine, which is bound by volatile ligands [26]. This is thought to be a mechanism by which male mice are able to extend the longevity of various scent markers.

To address the high variability in the concentration of urine samples among individuals, the concentrations of compounds in urine are reported after normalization to creatinine concentration [27]. Johnson measured creatinine concentration in human urine using an enzymatic method [25]. Lee et al. [28] used the derivatization method of Johnson for TMA and TMAO quantification and added a second protocol for creatinine quantification through LC-ESI-MS analysis. Both methods required urine volumes of 25 μL.

Our aim was to develop a sensitive and reproducible method, based on the methods of Johnson [25] and Lee et al. [28], that would allow quantification of TMA, TMAO and creatinine from only a drop of mouse urine. Lee et al. [28] included a chromatographic separation step which we chose to include because of the high protein concentration in male mouse urine, as mentioned above. To improve analyte separation, selectivity and sensitivity a modified gradient and a C_18_ column were used. This column is longer and has a smaller pore size than that used by Lee et al. [28]. We used a flow rate of 200 μL/min, selective ion monitoring (SIM) and a different instrumentation set-up, which are described below. A further modification of the method of Lee et al. [28] was the use of ^2^H_9_-TMA and ^2^H_9_-TMAO, as the internal standards for TMA and TMAO, instead of terfenadine. The method we describe was validated according to Guidelines for Bioanalytical Method Validation of the US FDA [29] and of the European Medicines Agency (EMEA) [30].

### Materials

TMA HCl (98% purity), creatinine (99% purity) and ethyl bromoacetate (98% purity) were obtained from Fischer Scientific (Loughborough, Leicestershire, UK). TMAO, acetaminophen, water (HPLC grade), formic acid (98% purity), ammonium hydroxide (99% purity) and ammonium formate were purchased from Sigma-Aldrich (Gillingham, Dorset, UK). Trimethyl-d9-amine HCl (^2^H_9_-TMA) was obtained from Qmx Laboratories (Thaxted, Essex, UK). Trimethyl-d9-amine *N*-oxide (98% purity) (^2^H_9_-TMAO) was purchased from Cambridge Isotope (Andover, MA, USA). Acetonitrile Hipersolv chroma, isocratic for HPLC, and anion-cation exchange (ACE) C_18_ (2.1 mm x 150 mm, 3 μm) column were purchased from VWR International (Lutterworth, Leicestershire, UK).

### Urine sample collection

Although metabolic cages are sometimes used for a continuous and complete 24-h urine collection, housing in metabolic cages can cause stress to animals, evaporation of urine and contamination of urine with food and/or feces. Male and female C57BL/6J mice were bred in-house at UCL. Male and female CD-1 mice were purchased from Charles River (Margate, Kent, UK) and allowed to acclimatize for 14 days before collection of urine. Mice were housed in an environmentally controlled room with a 12-h light/dark cycle and fed on a standard chow diet (Teklad Global 18% Protein Rodent Diet, Harlan Laboratories, Inc., Madison, WI, USA) with free access to water. Mice were 10 weeks old at the time of urine collection. Urine was obtained by gently holding a mouse and allowing it to urinate onto Saran wrap; drops of urine were collected using a micropipette [31]. Samples were collected between 10 a.m. and 12 noon (a.m. samples) or 2 p.m and 4 p.m (p.m. samples) and were stored at −80 °C until use. Animal procedures were carried out in accordance with the UK Animal Scientific Procedures Act and with local ethics committee approval and appropriate UK Home Office Licenses.

### Preparation of standards and urine samples for TMA and TMAO analysis

Working standards contained both TMA and TMAO, each at 7.8, 15.6, 31.3, 62.5, 125, 250 and 500 ng/μL in water. For aqueous standard solutions, 20 μL of each TMA-TMAO working standard was mixed with 5 μL of water. Urine samples were thawed to room temperature and 5 μL mixed with 20 μL of water. Derivatization reaction of TMA was as described by Johnson [25]. Briefly, to 25 μL of the prepared aqueous standards and urine samples, 1 μL ^2^H_9_-TMA (1 μg/μL), 2 μL of ^2^H_9_-TMAO (1 μg/μL), 1 μL of concentrated ammonia and 30 μL of ethyl bromoacetate (20 μg/μL in acetonitrile) were added. Samples were incubated for 30 min at room temperature. The reaction was stopped by the addition of 1 mL of 50% acetonitrile, 0.025% formic acid in water. Samples were centrifuged at 15,700 × *g* for 3 min at room temperature. Supernatant (100 μL) was diluted to a final volume of 1 mL with 50% acetonitrile, 0.025% formic acid in water. Concentrations of both derivatized TMA and TMAO in these aqueous standards were 15, 30, 59, 118, 236, 472 and 944 pg/μL. Twenty μL of an aqueous standard or of a urine sample were injected onto a C_18_ column.

### Preparation of standards and urine samples for creatinine analysis

Stock solutions of creatinine (1 μg/μL) and acetaminophen (20 μg/μL) were prepared in water and ethanol, respectively, and stored at −20 °C. Working standards of creatinine (0.24, 0.98, 3.91, 15.62, 62.5, 250 and 1000 ng/μL) were prepared in 0.1% formic acid in water. To 16 μL of each working standard of creatinine was added 1 μL of acetaminophen (20 μg/μL in ethanol) and 983 μL of 0.1% formic acid. Final concentrations of creatinine standards were 3.9, 15.6, 62.5, 250, 1000, 4000 and 16000 pg/μL. Urine was thawed to room temperature and 1 μL of acetaminophen IS (20 μg/μL in ethanol) added to 1 μL of urine followed by addition of 998 μL of 0.1% formic acid. Twenty μL of an aqueous creatinine standard or of a urine sample were injected onto a C_18_ column. Derivatization of TMA with ethyl bromoacetate affects creatinine and therefore creatinine is measured subsequent to the measurement of TMA and TMAO.

### Preparation of calibration standards

TMA-TMAO calibration standards were prepared in a similar fashion to aqueous standards, except that 20 μL of each TMA-TMAO working standard was added to 5 μL of mouse urine instead of water. The amount of TMA and TMAO in each calibration standard is a sum of the amount of TMA-TMAO in the working standard used and the endogenous amounts of TMA-TMAO in urine. Creatinine calibration standards were prepared by adding 1 μL of mouse urine to 16 μL of each creatinine working standard, followed by the addition of 1 μL of acetaminophen (20 μg/μL in ethanol) and 982 μL 0.1% formic acid. The amount of creatinine in each calibration standard is a sum of the amount of creatinine in the working standard used and the endogenous amount of creatinine in the urine sample. To determine the endogenous amounts of TMA, TMAO and creatinine in samples of urine used for the preparation of these calibration standards, a blank urine (unspiked) sample was also analyzed by LC–MS along with calibration standards.

### Preparation of quality control standards

Intra- and inter-day accuracies and precisions of quantification were determined using quality control (QC) standards (QCs) for TMA, TMAO and creatinine: (1) LLOQQC (Lowest Limit of Quantification Quality Control); (2) LQC (Low Quality Control); (3) MQC (Medium Quality Control) and (4) HQC (High Quality Control). The four QCs for TMA-TMAO were 15 pg/μL (LLOQQC), 45 pg/μL (LQC), 400 pg/μL (MQC) and 750 pg/μL (HQC) and for creatinine were 4 pg/μL (LLOQQC), 12 pg/μL (LQC), 6400 pg/μL (MQC) and 12000 pg/μL (HQC).

### Liquid chromatography mass spectrometry (LC–MS)

Capillary LC–MS (cap-LC–MS) analysis was performed using an Accela Thermo LC system (Thermo Scientific, UK) comprised of an Accela autosampler, vacuum degasser and pump system. The LC eluate was directed into the electrospray source, which was interfaced to a Finnigan linear ion trap LTQ mass spectrometer (Thermo Scientific, UK). Twenty μL of sample was injected through a 20-μL loop connected to an ACE C_18_ column maintained at 30 °C. The sample tray temperature was kept at 4 °C. The mobile phases were 5 mM ammonium formate buffer, pH 6.0, in water (mobile phase A) and 5 mM ammonium formate buffer, pH 6.0, in 90% acetonitrile (mobile phase B). Buffers were adjusted with formic acid to pH 6, filtered through 0.22-μm nylon membrane, degassed and stored at ambient temperature. LC was performed at a flow rate of 200 μL/min with gradient set up: 5% mobile phase A and 95% mobile phase B, changed to 95% mobile phase A and 5% mobile phase B over a period of 5 min. The eluate was directed into the LTQ mass spectrometer, which was operated in a positive-ion ESI mode, with seven scan events: full ESI scan followed by six selected ion monitoring scans (SIMs) over a 5-min LC run time. The ESI source was operated using the settings: spray voltage 4.5 kV, capillary temperature 220 °C, sheath gas 40 and auxiliary gas 10. The *m*/*z* range was scanned from 50 to 200, and centroid data were collected. SIMs were programmed to monitor derivatized-TMA (*m*/*z* 146) and derivatized-^2^H_9_-TMA (*m*/*z* 155); TMAO (*m*/*z* 76) and ^2^H_9_-TMAO (*m*/*z* 85); creatinine (*m*/*z* 114) and acetaminophen (*m*/*z* 152), respectively. Both the MS and SIMs scans consisted of three averaged microscans, each with a maximum injection time of 200 ms.

Between each urine sample injection, 20 μL of propan-2-ol:methanol (1:1, v/v) was injected at isocratic condition of 95% mobile phase B for a period of 5 min. The injection needle was washed 5 times with 100 μL of the same solvent. This was followed by 20 μL 0.1% formic acid in methanol on the C_18_ column using the LC gradient as for urine samples, to recondition the C_18_ column and to avoid any carry over of sample to the next injection.

### Data analysis

Data acquisition and processing was performed using Thermo Xcalibur Version 2.2 software (Thermo Scientific, UK). Seven-point calibration curves were constructed for the concentration range 15–944 pg/μL for TMA and TMAO and 3.9–16000 pg/μL for creatinine, along with a blank urine sample. Peak area ratios of analyte to its internal standard (IS) were calculated as a function ratio of analyte concentration. Back calculations were performed from these curves to determine the concentration of analytes. Calibration curves and lower and upper limits of quantification (LLOQ or ULOQ) were according to the FDA guidance [29].

### Method validation

The method was validated for analyte separation, selectivity, sensitivity, limit of detection (LOD), limit of quantification (LOQ), carry-over effect, system suitability, accuracy, precision, dilution integrity, ruggedness, robustness, matrix effect and stability as described in Supplementary data.

### Calculations and statistics

Endogenous analyte concentrations in urine used to prepare the calibration standards were determined and subtracted from the values determined in all the validation protocols that used QCs, in order to calculate the analyte concentration observed or recovered and to compare with the analyte concentration added during the preparation of the standard. Unknown amounts of TMA, TMAO and creatinine in mouse urine samples were quantified by reference to aqueous standard curves prepared as described in Sections Preparation of standards and urine samples for TMA and TMAO analysis and Preparation of standards and urine samples for creatinine analysis. Results are reported as mean ± standard error of the mean. P < 0.05 was selected for statistical significance. Statistical analyses were performed with GraphPad Prism Version 6.05 (GraphPad Software Inc., La Jolla, CA, USA).

## Chromatograms of analyte standards and TMA, TMAO and creatinine in mouse urine

**Fig. 1:**
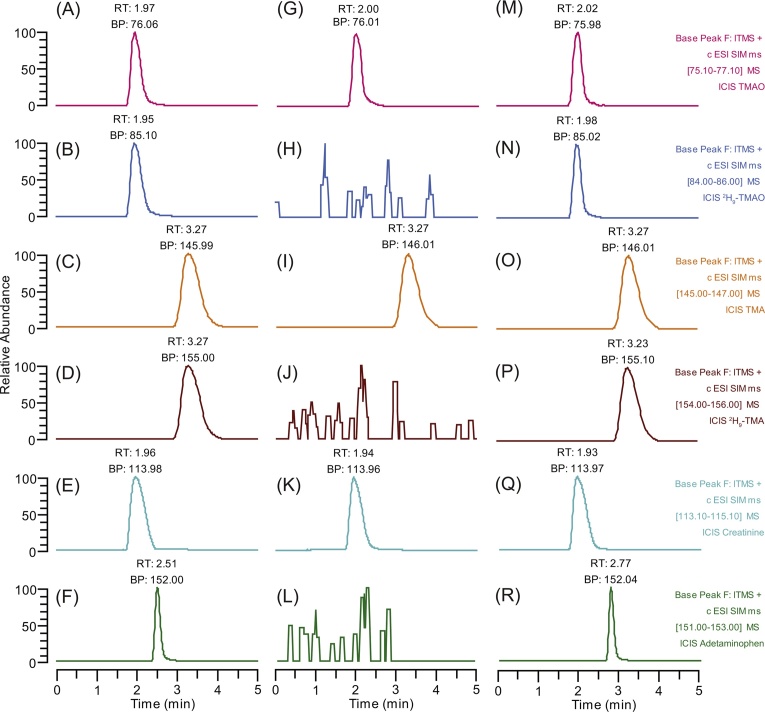
Reconstructed ion chromatograms (RICs) for pure standards (A-F), urine (G-L) and urine spiked with standards (M-R). RIC of SIM at *m*/*z* 76, corresponding to TMAO (A); RIC of SIM at *m*/*z* 85, corresponding to ^2^H_9_-TMAO (B); RIC of SIM at *m*/*z* 146, corresponding to derivatized TMA (C); RIC of SIM at *m*/*z* 155, corresponding to derivatized-^2^H_9_-TMA (D); RIC of SIM at *m*/*z* 114, corresponding to creatinine (E); RIC of SIM at *m*/*z* 152, corresponding to acetaminophen (F). RICs of urine for SIMs at *m*/*z* 76, endogenous TMAO (G), *m*/*z* 146, endogenous derivatized TMA (I) and *m*/*z* 114, endogenous creatinine (K); and RICs of a urine sample spiked with TMAO, ^2^H_9_-TMAO, TMA, ^2^H_9_-TMA, creatinine and acetaminophen for SIMs at *m*/*z* 76 (M), *m*/*z* 85 (N), *m*/*z* 146 (O), *m*/*z* 155 (P), *m*/*z* 114 (Q) and *m*/*z* 152 (R), respectively.

ESI settings, LC separation and optimization are described in Supplementary data. Pure standards of TMAO, ^2^H_9_-TMAO, derivatized TMA, derivatized-^2^H_9_-TMA, creatinine and acetaminophen were injected on the C_18_ column. Deuterium-labeled TMA and TMAO showed the same physico-chemical properties as their light analogs when injected on the C_18_ column. Reconstructed-ion chromatograms (RICs) for SIMs at *m*/*z* 76, 85, 146 155, 114 and 152 showed chromatographic peaks at retention times of 1.97, 1.95, 3.27, 3.27, 1.96 and 2.51 min, corresponding to TMAO, ^2^H_9_-TMAO, derivatized-TMA, derivatized-^2^H_9_-TMA, creatinine and acetaminophen, respectively (Fig. 1A–F).

Mouse urine contains endogenous TMA, TMAO and creatinine. When unspiked mouse urine was analyzed, RICs for SIMs at *m*/*z* 76, 146 and 114 showed three chromatographic peaks at 2.00, 3.27, and 1.94 min, corresponding to the retention times of TMAO, derivatized TMA and creatinine (Fig. 1G, I, K). As expected RICs for SIMs at *m*/*z* 85, 155, 152, corresponding to ^2^H_9_-TMAO, ^2^H_9_-TMA and acetaminophen, did not generate chromatographic peaks in mouse urine (Fig. 1H, J, L). RIC chromatograms for SIMs at *m*/*z* 76, 146 and 114 of the urine samples and the pure standards generated chromatographic peaks with the same retention times. Urine samples from both male and female mice were analyzed from two strains, C57BL/6J and CD-1. Samples collected in the morning (C57BL/6J: male, n = 5, female, n = 5; CD-1: male, n = 5, female, n = 6) and afternoon (C57BL/6J: male, n = 5, female, n = 5; CD-1: male, n = 5, female, n = 6) were analyzed for each mouse. No significant interfering chromatographic peaks or co-eluting chromatographic peaks at the retention times corresponding to derivatized TMA, TMAO and creatinine were present in any of the urine samples. Mouse urine spiked with standards, RICs for SIMs at *m*/*z* 76, 85, 146, 155, 114 and 152 showed chromatographic peaks at retention times of 2.02, 1.98, 3.27, 3.23, 1.93 and 2.77 min, corresponding to TMAO, ^2^H_9_-TMAO, derivatized-TMA, derivatized-^2^H_9_-TMA, creatinine and acetaminophen, respectively (Fig. 1M–R).

The chromatographic separation, together with SIM mass detection, gave an increased selectivity and sensitivity for the analysis of derivatized TMA, TMAO and creatinine and their isotope-labeled and IS analogs, suggesting there are no other endogenous interfering components in the urine samples. Therefore, the established LC-SIMs method has specificity and selectivity for the determination of TMA, TMAO and creatinine in mouse urine.

Our method does not require sample pre-treatment, such as solid-phase extraction. Despite the absence of this step there was no evidence of urine metabolites interfering with the quantification of TMA, TMAO or creatinine. The separation step on the C_18_ column and the selectivity of the MS using SIMs obviates the need for sample clean-up.

## Sensitivity and linearity

Table 1 lists the measured linearity of the calibration curves for TMA (15–944 pg/μL), TMAO (15–944 pg/μL) and creatinine (3.9–16000 pg/μL). Because endogenous TMA, TMAO and creatinine were detected in all mouse urine samples, ^2^H_9_-TMA, ^2^H_9_-TMAO and acetaminophen were chosen as the appropriate IS for the cap-LC-SIMs analysis. In this way, the influence of endogenous TMA, TMAO and creatinine on accurate quantification was minimized. Correlation coefficients (R^2^) of linear regression were ≥0.991 for all analytes in urine matrix, indicating a good quantitative relationship between the MS responses and the analyte concentrations. Using five replicates of standards, the accuracy was estimated as 92.36–103.46% and the precision was in the range 0.71–10.53%. Since no blank matrix mouse urine sample exists that is free of endogenous TMA, TMAO and creatinine, water, which is non-matrix solution, was used as a substitute matrix in preparing the aqueous standard curve (Table 1). The curves demonstrated good linearity and, therefore, were used to quantify TMA, TMAO and creatinine in urine.

**Table 1:** Limit of Detection (LOD), Limit of Quantification (LOQ) and linearity by cap-LC-ESI-MS-SIMs after alkylation of TMA.

Compound	LOD	LOQ	Aqueous Standards			Mouse Urine			
			Linearity (As/Ai)	SD of slope	R^2^	Linearity (As/Ai)	SD of slope	SD of Intercept	R^2^
TMA	28 pg/mL	84 pg/mL	0.01737C	0.000193	0.9992	0.01708C + 0.9454	0.000265	0.0279	0.9991
TMAO	115 pg/mL	345 pg/mL	0.00173C	0.000051	0.9999	0.00194C + 0.0081	0.000057	0.0051	0.9975
Creatinine	1 ng/mL	3 ng/mL	0.00023C	0.000020	0.9996	0.00019C + 0.0891	0.000018	0.0075	0.9910

As/Ai: peak area ratio of an analyte to the IS; C: concentration in pg/μL; SD: Standard Deviation.

The LOD and LOQ values shown in Table 1 are the on-column concentrations of TMA, TMAO and creatinine. All analytes showed dynamic range of >1000 for the MS response, which covered the biological concentration ranges for all analytes detected in mouse urine.

## Assay accuracy and precision

To determine the accuracy of quantification, the analytical recoveries were measured by cap-LC-SIMs using the IS compound spiking-in approach for TMA, TMAO and creatinine. Intra-day accuracy was evaluated from six analytical replicates prepared and analyzed by LC–MS. Table 2 shows the analytical recovery of TMA, TMAO and creatinine. All intra-day recoveries were between 96.49% and 105.65%. The precision of quantification was evaluated as coefficients of variation (CVs) between the samples. Intra-day precision was within 6.69%. Inter-day accuracy and precision was evaluated from six analytical replicates prepared and analyzed by LC–MS on four different days. Table 2 shows the inter-day accuracy was between 95.73% and 104.64% and precision was within 6.18%.

**Table 2:** Intra- and inter-day reproducibility and accuracy of LC-ESI-SIMs analysis for TMA, TMAO and creatinine.

Analyte	QC ID	Added Concentration (pg/μl)	Intra-day				Inter-day			
			n	Observed Concentration (pg/μl) (Mean ± SD)	% Accuracy	%CV	n	Observed Concentration (pg/μl) (Mean ± SD)	% Accuracy	%CV
TMA	LLOQQC	15	6	15.48 ± 0.50	103.24	3.25	24	14.85 ± 0.38	99.05	2.58
	LQC	45		44.48 ± 0.37	98.86	0.85		46.14 ± 0.79	102.54	1.72
	MQC	400		407.36 ± 9.44	101.84	2.32		391.67 ± 11.97	97.92	3.06
	HQC	750		723.67 ± 23.34	96.49	3.23		759.05 ± 23.06	101.21	3.04

TMAO	LLOQQC	15	6	14.91 ± 0.33	99.44	2.23	24	15.69 ± 0.97	104.64	6.18
	LQC	45		45.98 ± 3.07	102.18	6.69		45.83 ± 0.56	101.85	1.23
	MQC	400		387.29 ± 4.54	96.82	1.17		382.92 ± 16.38	95.73	4.28
	HQC	750		756.15 ± 23.50	100.82	3.11		770.85 ± 14.39	102.78	1.87

Creatinine	LLOQQC	4	6	4.22 ± 0.19	105.65	4.55	24	3.91 ± 0.11	97.87	2.91
	LQC	12		11.95 ± 0.11	99.63	0.99		11.86 ± 0.21	99.13	1.79
	MQC	6400		6536.96 ± 102.09	102.14	1.56		6447.36 ± 151.07	100.74	2.34
	HQC	12000		11582.40 ± 262.21	96.52	2.26		12402.19 ± 187.25	103.35	1.51

Validation of analyte carry-over effect, system suitability, dilution integrity, ruggedness, robustness, matrix effect and stability are as described in Supplementary data.

## Biological data

The concentrations of TMA and TMAO were normalized to that of creatinine, to account for any variations in the concentration of urine. FMO3 is the protein that converts TMA to TMAO in the liver. The expression of *Fmo3* is under the control of sex steroids [32]. In male mice, the expression of the *Fmo3* gene is switched off in liver at ∼6 weeks of age [33], [34]. The consequent lack of FMO3 activity in the liver of 10-week-old male mice is reflected in the data presented in Table 3. Male mice of both strains analyzed excrete in the morning 5.5 (C57BL/6J) and 4 (CD-1) times as much TMA (mM)/creatinine (mM) than do female mice. Conversely, female mice, which continue to express the *Fmo3* gene into adulthood, excrete approximately 4 times more TMAO (mM)/creatinine (mM) than do adult males. The total TMA, i.e., TMA + TMAO, excreted is very similar in male and female mice from the same strain. However, C57BL/6J mice excrete far greater amounts than do CD-1 mice of TMA (mM)/creatinine (mM) (male) and TMAO (mM)/creatinine (mM) (female).

**Table 3:** Concentrations of TMA and TMAO in urine collected at different times of day from male and female C57BL/6J and CD-1 mice.

		C57BL/6J		CD1	
		Morning	Afternoon	Morning	Afternoon
TMA (mM)/Creatinine (mM)	Male	3.095 ± 0.519	0.623 ± 0.065 ^(^^)^	0.404 ± 0.037 ^(&&&)^	0.703 ± 0.077 ^(^^)^
	Female	0.552 ± 0.066 ^(**)^	0.225 ± 0.036 ^(***/^^)^	0.104 ± 0.006 ^(****/&&&&)^	0.074 ± 0.013 ^(****/&&)^
TMAO (mM)/Creatinine (mM)	Male	0.704 ± 0.030	0.170 ± 0.007 ^(^^^^)^	0.180 ± 0.015 ^(&&&&)^	0.166 ± 0.013
	Female	2.729 ± 0.201 ^(****)^	1.338 ± 0.285 ^(**/^^)^	0.657 ± 0.059 ^(****/&&&&)^	0.483 ± 0.091 ^(*/&)^
TMAO:TMA	Male	0.247 ± 0.042	0.280 ± 0.024	0.453 ± 0.041 ^(&)^	0.250 ± 0.034 ^(^^)^
	Female	5.064 ± 0.471 ^(****)^	5.865 ± 0.455 ^(****)^	6.302 ± 0.249 ^(****/&)^	6.519 ± 0.302 ^(****)^

Values are mean ± SEM, n = 5–6. As indicated, significant differences were observed between male and female mice of the same strain at the same time of day (*), morning and afternoon for mice of the same strain and sex (^) and different mouse strains of the same sex and at the same time of day (&). * = p < 0.05, ** = p < 0.01, *** = p < 0.001, **** = p < 0.0001, ^^ = p < 0.01, ^^^^ = p < 0.0001, & = p < 0.05, && = p < 0.01, &&& = p < 0.001, and &&&& = p < 0.0001.

Our results demonstrate that the design of biological experiments reliant on the use of urinary TMA or TMAO as biomarkers in mouse models must consider gender, strain and the time of day urine samples are collected. The method described would also be suitable for quantification of TMA, TMAO and creatinine in clinical studies, including diagnosis of the disorder trimethylaminuria.

## Conclusion

We have developed a sensitive method, combining chemical derivatization with ethyl bromoacetate and cap LC-SIMs, for the targeted analysis of TMA, TMAO and creatinine and their IS in mouse urine. All analyte identification was achieved based on chromatographic separation with unique *m*/*z* values. The cap LC–SIMs was used for accurate quantification and we also performed scans for structural confirmation of the analytes. The method obviates the need for extra purification steps and allows all three analytes to be quantified rapidly (within 5 min) in a urine volume as small as 6 μL. The selectivity, sensitivity, precision, accuracy, robustness and reproducibility of the method demonstrate that it is well suited for quantification of TMA, TMAO and creatinine, particularly in cases where sample volume is limited, such as the urine of mice, a species that is often used in research and preclinical studies. The results demonstrate that the design of biological experiments reliant on the use of TMA or TMAO as biomarkers in mouse models must consider gender, strain and the time of day urine samples are collected. The method would also be suitable for quantification of TMA, TMAO and creatinine in clinical studies, including diagnosis of the disorder trimethylaminuria.

## Appendix A Supplementary data

Supplementary data summarises the methods used for validation, derivatisation reactions and chemical structures of all analytes, capillary ESI–MS for derivatized TMA, TMAO, creatinine and acetaminophen to establish their mass spectrometry signal under ESI conditions, RIC for blank samples, and validation results for dilution integrity, ruggedness, robustness, matrix factor and stability tests.
