# Supplementary material for: A highly sensitive liquid chromatography electrospray ionization mass spectrometry method for quantification of TMA, TMAO and creatinine in mouse urine
Source: MethodsX. 2017 Sep 28;4:310–9. doi: 10.1016/j.mex.2017.09.004 (PMC5643081; doi:10.1016/j.mex.2017.09.004)
Supplement: Supplementary file 1 [file mmc1.docx]

**Supplementary data**

**Title: A highly sensitive liquid chromatography electrospray ionization mass spectrometry method for quantification of TMA, TMAO and creatinine in mouse urine**

Sunil Veeravalli^a^, Kersti Karu^b^, Ian R. Phillips^a,c^, and Elizabeth A Shephard^a,*^

^a^ *Institute of Structural and Molecular Biology, University College London, London, UK*

^b^ *Mass Spectrometry Facility, Department of Chemistry, University College London, London, UK*

^c^ *School of Biological and Chemical Sciences, Queen Mary University of London, London, UK*

Corresponding author:

e.shephard@ucl.ac.uk

+44(20)76792321

**1. Experimental**

**1.1 Chromatographic separation,** **selectivity and sensitivity of the analytes**

Chromatographic separation of high-purity analytical standards of TMA, TMAO, creatinine and their IS ^2^H­­_9_-TMA, ^2^H­­_9_-TMAO and acetaminophen (100 pg/µL each) on the C_18_ column was first established. Mouse urine contains many unknown metabolites. Therefore, we analysed water, urine and urine spiked with TMA, TMAO, ^2^H­­_9_-TMA, ^2^H­­_9_-TMAO, creatinine and acetaminophen individually to establish the elution time, selectivity and separation of the target analytes TMA, TMAO and creatinine from unknown metabolites on the C_18_ column.

**1.2 Limit of detection (LOD) and limit of quantification (LOQ)**

Working standards of TMA, TMAO and creatinine were diluted stepwise with 50% acetonitrile, 0.025% formic acid in water, with a dilution factor of 3. These standard solutions were subjected to ethyl bromoacetate derivatization, followed by LC-ESI-SIM analysis. The signal-to-noise ratios were used to determine the LODs and LOQs. The lower LODs and lower LOQs were defined as on-column amounts that gave rise to peaks with signal-to-noise ratios (S/N) of 3 and 10, respectively, according to the United States Pharmacopeia guidelines [1].

**1.3 Carry-over effect**

The carry-over effect was evaluated by injecting the TMA, TMAO and creatinine calibration standards at ULOQ level, which was immediately followed by three blank injections of mobile phase A. The carry-over was considered to be acceptable if the area counts of the analytes and IS for the blank injections were not more than 20% and 5%, respectively, compared to the area counts generated from LLOQ standard of the calibration curve.

**1.4 System suitability**

Six consecutive injections of aqueous standards of TMA-TMAO and creatinine were performed and the variation in the analyte signal between the replicates was calculated to measure system suitability.

**1.5 Assay accuracy and precision**

For intra-day accuracy and precision, six replicates of TMA-TMAO and creatinine quality control standards were run on the same day. The precision of the quantification was measured as the intra-day coefficient of variation (CV). The accuracy of quantification was measured as the intra-day analytical recovery of the analytes in quality control standards used. Accuracy is a percent recovery of analyte calculated as (mean observed concentration/spiked concentration) x 100%. Mean observed concentration was measured by subtracting the concentration of endogenous analyte in urine from the total concentration of analytes in quality controls, both determined from a calibration curve. The spike-in amounts correspond to 15, 45, 400 and 750 pg/µL for TMA and TMAO, and 4, 12, 6,400 and 12,000 pg/µL for creatinine.

The inter-day precision and accuracy of quantification were measured by analysing the TMA-TMAO and creatinine quality control standards on four consecutive days. Precision is expressed as percentage coefficient of variation (%CV), which should not exceed 15% for QC samples except for the LLOQQC, which should not exceed 20%. The accuracy percentage should be within ±15% (i.e., 85-115%) for QC samples except for LLOQQC, which should be within ±20% (i.e., 80-120%).

**1.6 Dilution integrity of the quantification of TMA, TMAO and creatinine by LC-ESI-SIMs**

The dilution integrity of the assay was validated with a dilution quality control sample (DQC), which was prepared by spiking a urine sample with an analyte concentration of TMA, TMAO or creatinine 1.5 times greater than the ULOQ (Upper Limit of Quantification). This DQC sample was diluted to 1/5^th^ and 1/10^th^ with water. Six replicates for the 1/5^th^ and 1/10^th^ dilutions, along with freshly spiked calibration standards LQC and HQC, were analysed on the LC-MS system. The generated data were then back calculated to measure the precision and accuracy of quantification for TMA, TMAO and creatinine. Dilution integrity is considered to be maintained if accuracy is within ±15% of nominal values and precision (%CV) is ≤15%. From this level of dilution integrity of our method, our chosen volumes of urine of 5µl and 1 µl were enough to measure TMA and TMAO, and creatinine, respectively, with this method.

**1.7 Ruggedness and robustness**

The ruggedness of the assay was measured with six replicates of TMA-TMAO and creatinine quality control standards analysed on the same column and LC-MS system by two different analysts and also on two different columns of the same make (ACE C_18_ columns), by a single analyst on the same LC-MS system. The ruggedness is considered met based on the linearity, reproducibility and precision and accuracy between the replicates.

To determine the robustness of the LC-MS method, the effect of flow rate was studied at 180 µL/min and 220 µL/min, instead of 200 µL/min; and the effect of column temperature at 25°C and 35°C, instead of 30°C. The intra-day coefficient of variation (CV) for six replicates of TMA-TMAO and creatinine LLOQQC and HQC standards were used to measure the robustness of the LC-MS analysis.

**1.8 Matrix factor**

The matrix factor (MF) was determined by injecting six replicates of TMA-TMAO and creatinine LLOQQC and HQC standards prepared by spiking into 5 µL of mouse urine or 5 µL of water. Following derivatization and subsequent LC- SIMs analysis, MF was calculated as the ratio of peak area of analytes in urine to their peak area observed in water. Normalized MF was calculated as a ratio of MF of the analyte to the MF of its IS.

**1.9 Stability of TMA, TMAO, creatinine and acetaminophen in mouse urine**

The stability of TMA, TMAO, creatinine and acetaminophen in mouse urine was determined by analysing six replicates of LQC and HQC quality control standards under a variety of storage and processing conditions, namely, short-term stability, freeze-thaw stability, the autosampler storage stability and long-term stability. Samples were considered stable if the deviation from the mean calculated concentration of freshly prepared quality control samples was within ±15%. The short-term stability was determined after exposure of the QC standards to room temperature for 6 h before sample preparation. Freeze-and-thaw stability was determined by three freeze and thaw cycles followed by LC-MS analysis. One cycle consisted of freezing the QC standards to -80°C for 24 h and then thawing them to room temperature. The autosampler storage stability was determined by storing the QC standards for 24 h under autosampler conditions (maintained at 4°C) before being analysed by LC-MS. The long-term stability was assessed after storage of the QC standards at -80°C for 4 weeks before LC-MS analysis. All stability exercises for QC standards were performed against freshly prepared calibration standards.

The stock and working standards of TMA, TMAO, creatinine and acetaminophen were also analysed for short- and long-term stability after storing at room temperature for 12 h and at refrigerator temperature (4°C) for 10 days, respectively. The stability was measured against freshly prepared aqueous standards.

**1.10 Direct infusion capillary ESI-MS analysis**

Here we describe the direct infusion ESI mass spectrometry experiment for TMA derivatized with ethyl bromoacetate, TMAO, creatinine and acetaminophen and their corresponding MS^2^ and MS^3^, which allow the identification and structural characterization of these analytes in mouse urine.

TMAO, derivatized TMA, creatinine and acetaminophen were analysed individually by a direct infusion electrospray ionisation (ESI) into a Finnigan LTQ (now Thermo Fisher, UK) linear ion-trap mass spectrometer. This was achieved by loading sample (200 μL) into a Hamilton syringe (Thames Restek UK Limited, Saunderton, Bucks, UK) and pumping the solution at a flow rate of 5 µL/min into the electrospray source. The ESI source was operated using the following settings: spray voltage 4 kV, capillary temperature 220 °C, sheath gas 10 and auxiliary gas 2. The *m/z* range was scanned from 50 to 200, and centroid data were collected. Other conditions were optimised automatically using the autotune function incorporated into the LTQ tune page. Manual acquisition was performed to record MS and MS^2^ (MS/MS) spectra. ESI full mass and MS^2^ spectra were recorded using a positive-ion mode. The collision energy setting was 45% (arbitrary units), the isolation width 2.0 units. MS, MS^2^ scans consisted of three averaged “microscans”, each with a maximum injection time of 200 ms.

For ESI infusion analysis, 100 pg/µL each of TMAO, creatinine and acetaminophen were prepared in 50% acetonitrile, 0.1% formic acid in water, whereas TMA was derivatized with ethyl bromoacetate, as described in the Experimental Section.

**2. Results and Discussion**

**2.1 Derivatization reaction and chemical structures of analytes**

For quantitative derivatization, TMA and ^2^H_9_-TMA were alkylated by ethyl bromoacetate, generating a quaternary ion [N(CH_3_)_3_CH_3_]^+^ (Figure S1).


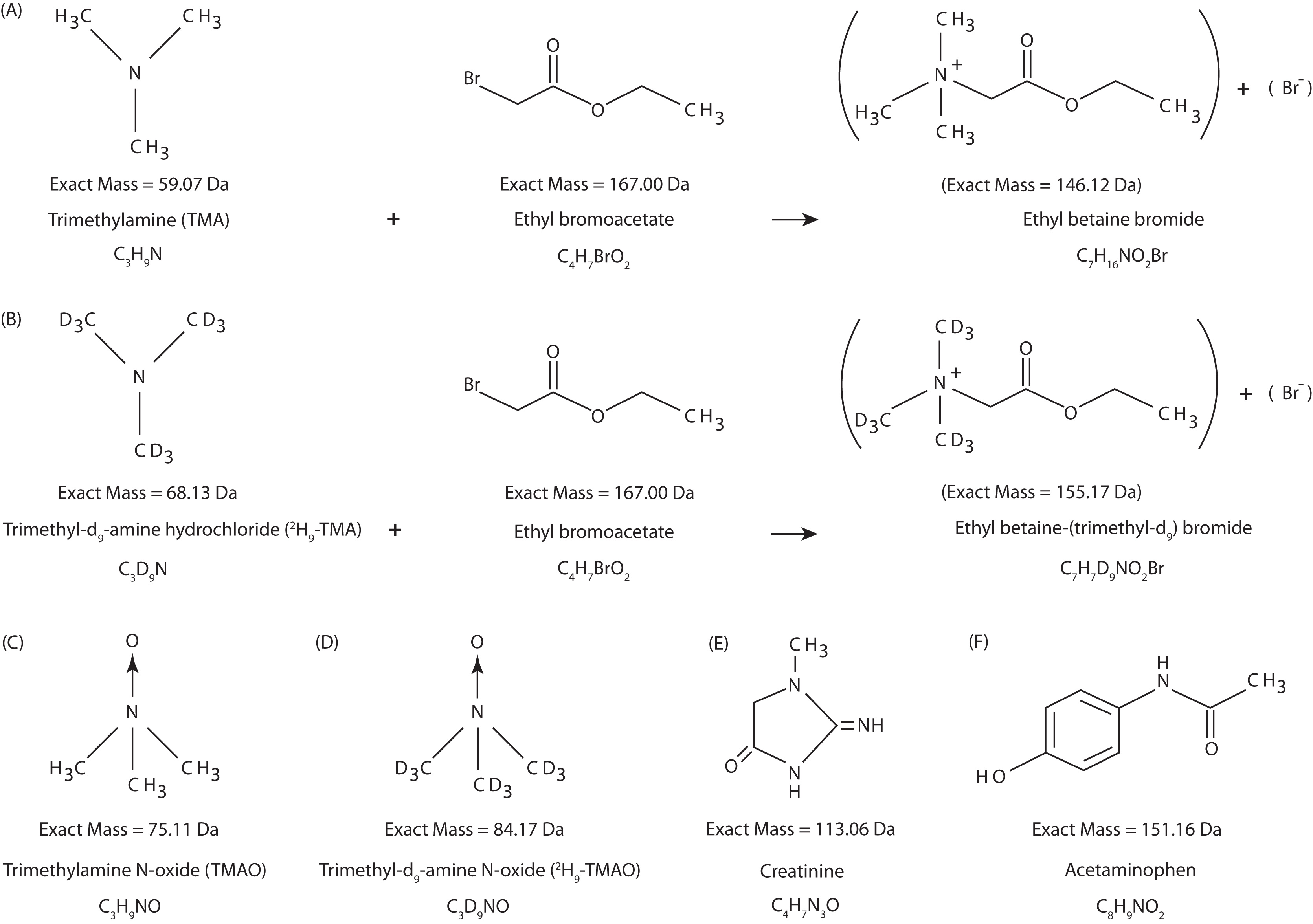


**Figure S1.** Derivatization reaction of (A) TMA and (B) ^2^H_9_-TMA with ethyl bromoacetate. The chemical structures of (C) TMAO, (D) ^2^H_9_-TMAO, (E) creatinine and (F) acetaminophen.

**2.2 Direct infusion ESI mass spectra**

The ESI mass spectrum shows a peak at *m/z* 146, corresponding to [M]^+^ ion of the derivatized TMA (Figure S2). However, there are several additional peaks present at *m/z* 59, 105, 145, 190, corresponding to components present in the reaction mixture. To confirm the identity of the derivatized TMA and TMAO, we performed MS^2^ and MS^3^ experiments.

[C_7_H_16_NO_2_]^+^

[C_4_H_7_O_2_Br+Na]^+^

Infusion mix

Infusion mix

**Figure S2.** ESI mass spectrum of TMA derivatized by alkylation with ethyl bromoacetate, in 50% acetonitrile in water, 0.025% formic acid. Note: the peak at *m/z* 146 corresponds to derivatized TMA, whereas those at *m/z* 59, 104, 144.9 and 190 correspond to other components of the reaction mixture.

When fragmented by MS^2^ derivatized TMA, corresponding to *m/z* 146, gives an intense [M-28]^+^ ion, corresponding to the loss of CO (Figure S3), which can be fragmented further by MS^3^ to give characteristic product ions at *m/z* 58 and 59. These ions were generated due to loss of C_4_H_7_O_2_ and C_4_H_8_O_2_ from the derivatized TMA (Figure S4).

**Figure S3.** MS^2^ (146→) spectrum of TMA derivatized with ethyl bromoacetate.

**Figure S4.** MS^3^ (146→118→) spectrum of TMA derivatized with ethyl bromoacetate.

The ESI mass spectrum of the derivatized ^2^H_9_-TMA shows a peak at *m/z* 155. The mass difference between derivatized TMA and derivatized deuterium-labeled TMA is 9 Da, owing to the replacement of the nine hydrogen atoms of TMA with deuterium. The essentially identical chemical properties of the heavy and light TMA means that they react with ethyl bromoacetate at a similar rate, giving essentially similar products, which, however, differ in mass. The mass difference allows the quantification of TMA by MS.

ESI mass spectra of light TMAO and heavy ^2^H_9_-TMAO showed peaks at *m/z* 76 (Figure S5) and *m/z* 85, corresponding to their respective [M]^+^ ions. MS^2^ fragmentation of [M]^+^ ions of *m/z* 76 generated two characteristic ions at *m/z* 58 and 59, corresponding respectively to the loss of water and an OH group (Figure S6).

F:

ITMS + c ESI Full ms [68.00-200.00]

70

80

90

100

110

120

130

140

150

160

170

180

190

200

m/z

0

5

10

15

20

25

30

35

40

45

50

55

60

65

70

75

80

85

90

95

100

Relative Abundance

76.2

77.2

196.2

**Figure S5.** ESI mass spectrum of TMAO infused into the ion-trap mass spectrometer. The concentration of TMAO was 100 pg/µL in 50% acetonitrile, 0.1% formic acid in water.

**Figure S6.** MS^2^ (76→) spectrum of TMAO.

ESI mass spectrum of creatinine showed a peak at *m/z* 114, corresponding to the [M+H]^+^ ion (Figure S7), whereas the ESI mass spectrum of acetaminophen generated a peak at *m/z* 152, corresponding to [M+H]^+^ (Figure S8).

**Figure S7.** ESI mass spectrum of creatinine infused into the ion-trap mass spectrometer. The concentration of creatinine was 100 pg/µL in 50% acetonitrile, 0.1% formic acid in water.

F:

ITMS + c ESI Full ms [68.00-200.00]

70

80

90

100

110

120

130

140

150

160

170

180

190

200

m/z

0

5

10

15

20

25

30

35

40

45

50

55

60

65

70

75

80

85

90

95

100

Relative Abundance

152.2

105.5

153.2

174.1

**Figure S8.** ESI mass spectrum of acetaminophen infused into the ion-trap mass spectrometer. The concentration of acetaminophen was 100 pg/µL in 50% acetonitrile, 0.1% formic acid in water.

**2.3 ESI mass spectra**

To quantify the amount of TMA, TMAO and creatinine present in mouse urine, we performed ethyl bromoacetate derivatization of TMA, followed by the cap-LC-ESI interfaced with a Thermo Finnigan linear ion-trap mass spectrometer. We utilized internal standards that were either stable-isotope-labeled analogs or structural analogs, which have identical physical and chemical properties to the analytes, to allow accurate quantification of the analytes in mouse urine. ESI is prone to matrix effects from metabolites present in urine, which can suppress or enhance recoveries of analytes during the sample preparation and LC-MS analysis. The IS used were ^2^H_9_-TMA for TMA, ^2^H_9_-TMAO for TMAO, and acetaminophen for creatinine. TMA was quantitatively alkylated by ethyl bromoacetate, generating the quaternary ion [N(CH_3_)_3_CH_3_]^+^ (Figure S1). This derivatization allows an increase of the ionization efficiency of TMA under electrospray conditions. The ESI mass spectrum showed a peak at *m/z* 146, corresponding to [M]^+^ ion of the derivatized-TMA (Figure S2). MS^2^ of the *m/z* 146 species showed a major fragment ion at *m/z* 118, corresponding to the loss of 28, corresponding to ethylene (Figure S3). The ESI mass spectrum of the derivatized ^2^H_9_-TMA shows a peak at *m/z* 155 (data not shown). The mass difference between derivatized TMA and derivatized deuterium-labeled TMA is 9 Da, corresponding to the nine deuterium atoms added to the TMA molecule.

The essentially identical chemical properties of the heavy and light TMA mean that they react with ethyl bromoacetate to give a similar product. The mass difference allows the quantification of TMA by MS. ESI mass spectra of light TMAO and heavy ^2^H_9_-TMAO showed a peak at *m/z* 76 (Figure S5) and *m/z* 85 (data not shown), respectively, corresponding to their [M]^+^ ions. ESI mass spectra of creatinine and acetaminophen showed a peak at *m/z* 114 (Figure S7), corresponding to [M+H]^+^ ion, and *m/z* 152 (Figure S8), corresponding to [M+H]^+^, respectively.

**2.4 Optimisation of the LC separation and MS**

The chromatographic separation of the derivatized TMA, TMAO, creatinine and acetaminophen was tested on several reverse-phase C_18_ columns using 0.1% formic acid in water (solvent A) and 0.1% formic acid in acetonitrile (solvent B) for binary gradient elution. Baseline separation was achieved for creatinine and acetaminophen, but not for the derivatized TMA and TMAO, even when using the optimal elution gradient conditions. Better separation of derivatized TMA, TMAO, creatinine and acetaminophen was achieved with an ACE C_18_ column with 5 mM ammonium formate buffer in water, pH 6 (mobile phase A) and 90% acetonitrile in 5 mM ammonium formate in water, pH 6 (mobile phase B), using a step gradient.

For mass detection, our preference was to perform mass analysis using full-scan mode and selective-ion-monitoring (SIM)-scan mode simultaneously, because of the fast scanning speed of the LQT mass spectrometer and also the use of stable-isotope or structural-analog IS for quantification. The identification of analytes was achieved based on chromatographic separation and their unique *m/z* values. Several LC-MS runs of blank samples (just water), pure standards, urine samples and urine samples spiked with standards were conducted to establish the robustness of the above cap-LC-MS analysis. Quantification at the SIM level was achieved by generating the reconstructed ion chromatograms (RICs) for each SIM corresponding to derivatized TMA (*m/z* 146) and ^2^H_9_-TMA (*m/z* 155), TMAO (*m/z* 76), ^2^H_9_-TMAO (*m/z* 85), creatinine (*m/z* 114) and acetaminophen (*m/z* 152). Figure S9 A to F shows the RICs for the SIMs when the blank water sample was injected on the C_18_ column. No TMA, TMAO, creatinine or acetaminophen were detected in the blank water.


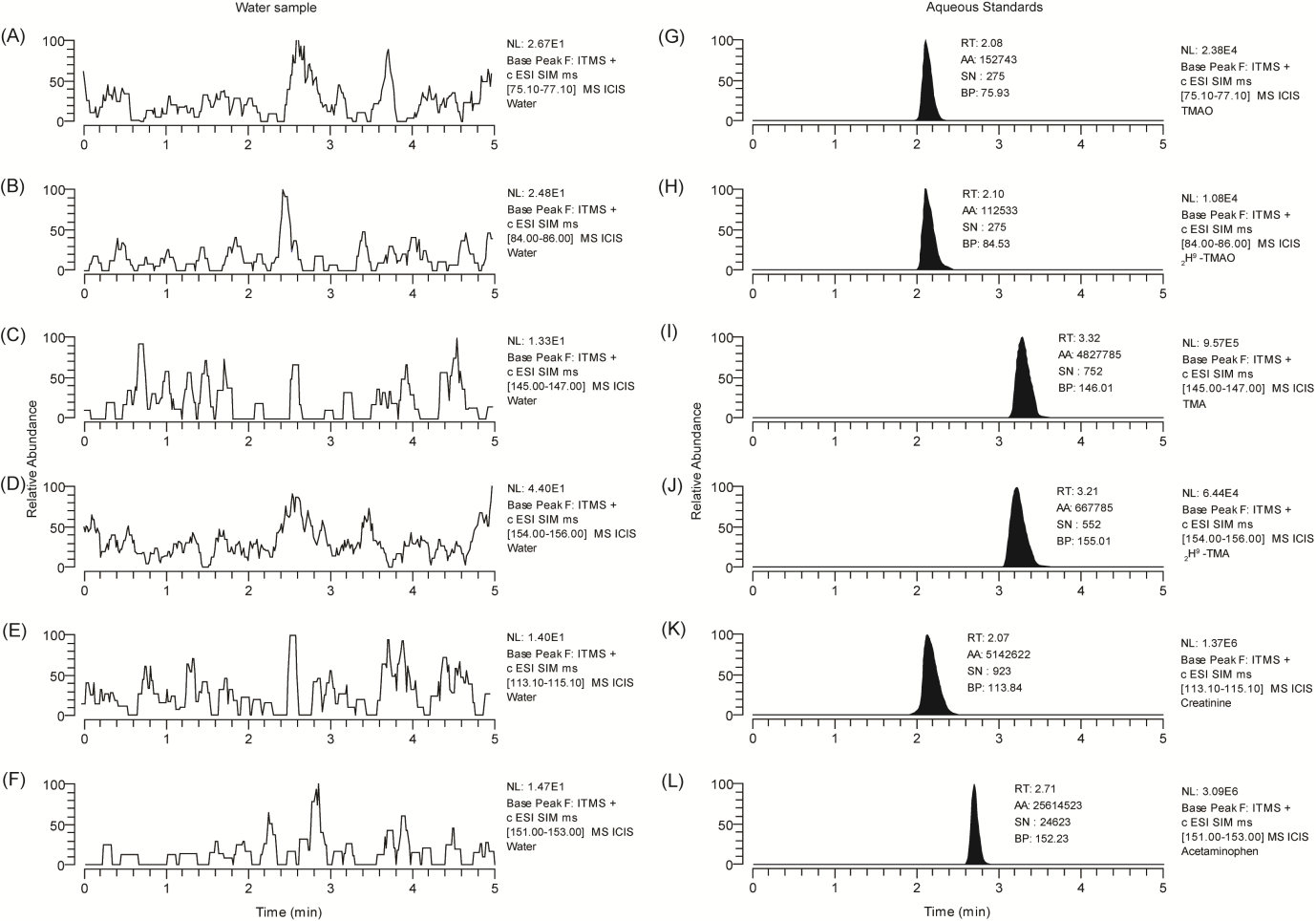


**Figure S9.** Reconstructed ion chromatograms (RICs) for blank samples. RIC of SIM at *m/z* 76, corresponding to TMAO (A); RIC SIM at *m/z* 85, corresponding to ^2^H_9_-TMAO (B); RIC of SIM at *m/z* 146, corresponding to derivatized TMA (C); RIC of SIM at *m/z* 155, corresponding to derivatized-^2^H_9_-TMA (D); RIC of SIM at *m/z* 114, corresponding to creatinine (E) and RIC of SIM at *m/z* 152, corresponding to acetaminophen (F).

**2.5 Carry-over effect**

Carry-over evaluation was performed to ensure that accuracy and precision of the method were not affected. This was estimated as the detection of an analyte signal in the blank sample, injected immediately after the highest concentration of standard (ULOQ). Negligible carry-over of ≤3% and ≤0.5% of LLOQ response was detected with TMAO and derivatized-^2^H_9_-TMA, respectively, in the blank sample. This carry-over was not found after wash and blank runs were included after analysis of each urine sample.

**2.6 Dilution integrity**

Dilution integrity was performed to determine whether the dilution of a sample has an effect on accuracy or precision. The accuracy and precision for 1/5th and 1/10th dilutions of derivatized TMA, TMAO and creatinine were calculated to be within the range 95.70% to 105.53% and 2.72% to 6.47%, respectively, and the values are shown in Table S1. Though the dilutions result in a difference in matrix content between QC samples and diluted samples, our results demonstrate that the dilution of the concentrated urine sample up to 10 times maintains the integrity of all analyte concentrations. Thus, our method is suitable for using the lower end of a calibration curve if an unknown sample analyte concentration falls in that range.

**Table S1:** Dilution integrity of TMA, TMAO and creatinine in mouse urine.

| **Analyte** | **Dilution** | **Added Concentration (pg/µl)** | **n** | **Observed Concentration (pg/µl) (Mean ± SD)** | **% Accuracy** | **% CV** |
| --- | --- | --- | --- | --- | --- | --- |
|  |  |  |  |  |  |  |
| TMA | 1/5 | 300 | 6 | 311.43 ± 12.92 | 103.81 | 4.15 |
|  | 1/10 | 150 |  | 158.31 ± 6.51 | 105.53 | 4.11 |
|  |  |  |  |  |  |  |
| TMAO | 1/5 | 300 | 6 | 287.09 ± 18.56 | 95.70 | 6.47 |
|  | 1/10 | 150 |  | 152.61 ± 5.18 | 101.74 | 3.40 |
|  |  |  |  |  |  |  |
| Creatinine | 1/5 | 4800 | 6 | 4916.16 ± 160.93 | 102.42 | 3.27 |
|  | 1/10 | 2400 |  | 2363.76 ± 64.32 | 98.49 | 2.72 |
|  |  |  |  |  |  |  |

**2.7 Ruggedness and robustness**

The ruggedness results (Table S2) show that the accuracy and precision of the derivatized TMA, TMAO and creatinine for the first analyst ranged from 96.12% to 105.97% and 1.63% to 5.35%, respectively, and for the second analyst ranged from 96.52% to 105.17% and 1.51% to 5.54%, respectively. When using two different C_18_ columns from the same manufacturer, derivatized TMA, TMAO and creatinine were quantified with an accuracy and precision ranging from 96.39% to 104.67% and 1.63% to 5.05%, respectively, for the first column and 94.38% to 105.22% and 0.93% to 5.86%, respectively, for the second column.

Robustness results shown in Table S3 are a comparison of concentrations of derivatized TMA, TMAO and creatinine that were detected at three different column temperatures and at three different flow rates. With a change in column temperature, the accuracy and precision of LLOQQC ranged from 95.48% to 106.62% and 0.14% to 7.70%, respectively. For HQC, the accuracy and precision ranged from 94.88% to 107.27% and 0.61% to 8.18%, respectively. With a change in the LC flow rate, the LLOQQC samples were quantified with the accuracy and precision ranging from 95.15% to 105.86% and 0.58% to 7.97%, respectively. For the HQC samples, the accuracy and precision was 97.13% to 106.99% and 0.12% to 7.51%, respectively. Both the ruggedness and robustness were found to be well within the acceptance limit of 15% in the precision measurement and 85% to 115% in the mean accuracy measurement.

**Table S2:** Ruggedness of the method tested by two different analysts and by using two different columns.

| **Analyte** | **Quality control standard** | **Analyte added (pg/µl)** | **n** |  | **Analyte recovered (pg/µl) (Mean ± SD)** | **% Accuracy** | **%CV** |  | **Analyte recovered (pg/µl) (Mean ± SD)** | **% Accuracy** | **%CV** |
| --- | --- | --- | --- | --- | --- | --- | --- | --- | --- | --- | --- |
|  |  |  |  |  | **Analyst 1** | | |  | **Analyst 2** | | |
|  |  |  |  |  |  |  |  |  |  |  |  |
| TMA | LLOQQC | 15 | 6 |  | 14.82 ± 0.29 | 98.83 | 1.97 |  | 15.42 ± 0.23 | 102.81 | 1.51 |
|  | LQC | 45 |  |  | 45.27 ± 1.35 | 100.59 | 2.97 |  | 44.48 ± 1.36 | 98.84 | 3.07 |
|  | MQC | 400 |  |  | 412.92 ± 19.20 | 103.23 | 4.65 |  | 414.16 ± 19.82 | 103.54 | 4.78 |
|  | HQC | 750 |  |  | 727.58 ± 26.49 | 97.01 | 3.64 |  | 759.23 ± 19.67 | 101.23 | 2.59 |
|  |  |  |  |  |  |  |  |  |  |  |  |
| TMAO | LLOQQC | 15 | 6 |  | 14.97 ± 0.76 | 99.78 | 5.05 |  | 14.48 ± 0.59 | 96.52 | 4.10 |
|  | LQC | 45 |  |  | 47.69 ± 1.90 | 105.97 | 3.99 |  | 45.91 ± 2.55 | 102.02 | 5.54 |
|  | MQC | 400 |  |  | 416.68 ± 9.58 | 104.17 | 2.30 |  | 420.68 ± 9.35 | 105.17 | 2.22 |
|  | HQC | 750 |  |  | 720.90 ± 38.54 | 96.12 | 5.35 |  | 747.23 ± 27.74 | 99.63 | 3.71 |
|  |  |  |  |  |  |  |  |  |  |  |  |
| Creatinine | LLOQQC | 4 | 6 |  | 4.09 ± 0.14 | 102.34 | 3.31 |  | 3.89 ± 0.17 | 97.31 | 4.43 |
|  | LQC | 12 |  |  | 11.75 ± 0.31 | 97.91 | 2.63 |  | 12.53 ± 0.42 | 104.38 | 3.38 |
|  | MQC | 6400 |  |  | 6724.48 ± 290.25 | 105.07 | 4.32 |  | 6426.24 ± 119.07 | 100.41 | 1.85 |
|  | HQC | 12000 |  |  | 12177.60 ± 198.84 | 101.48 | 1.63 |  | 11770.80 ± 608.35 | 98.09 | 5.17 |
|  |  |  |  |  |  |  |  |  |  |  |  |
|  |  |  |  |  | **Column 1** | | |  | **Column 2** | | |
|  |  |  |  |  |  |  |  |  |  |  |  |
| TMA | LLOQQC | 15 | 6 |  | 15.70 ± 0.64 | 104.67 | 4.11 |  | 14.96 ± 0.13 | 99.78 | 0.93 |
|  | LQC | 45 |  |  | 43.37 ± 1.58 | 96.39 | 3.65 |  | 45.61 ± 1.74 | 101.37 | 3.83 |
|  | MQC | 400 |  |  | 404.92 ± 12.87 | 101.23 | 3.18 |  | 377.51 ± 14.25 | 94.38 | 3.78 |
|  | HQC | 750 |  |  | 767.70 ± 36.40 | 102.36 | 4.74 |  | 789.13 ± 46.22 | 105.22 | 5.86 |
|  |  |  |  |  |  |  |  |  |  |  |  |
| TMAO | LLOQQC | 15 | 6 |  | 14.74 ± 0.24 | 98.32 | 1.63 |  | 15.12 ± 0.30 | 100.84 | 2.02 |
|  | LQC | 45 |  |  | 45.27 ± 1.29 | 100.62 | 2.87 |  | 44.42 ± 1.84 | 98.72 | 4.15 |
|  | MQC | 400 |  |  | 416.36 ± 21.04 | 104.09 | 5.05 |  | 407.64 ± 12.64 | 101.91 | 3.10 |
|  | HQC | 750 |  |  | 763.35 ± 17.17 | 101.78 | 2.25 |  | 780.22 ± 12.90 | 104.03 | 1.65 |
|  |  |  |  |  |  |  |  |  |  |  |  |
| Creatinine | LLOQQC | 4 | 6 |  | 4.14 ± 0.14 | 103.51 | 3.49 |  | 4.01 ± 0.10 | 100.31 | 2.74 |
|  | LQC | 12 |  |  | 11.93 ± 0.53 | 99.47 | 4.45 |  | 12.35 ± 0.55 | 102.96 | 4.49 |
|  | MQC | 6400 |  |  | 6403.21 ± 164.10 | 100.05 | 2.56 |  | 6349.44 ± 82.03 | 99.21 | 1.29 |
|  | HQC | 12000 |  |  | 12351.65 ± 469.12 | 102.93 | 3.80 |  | 12292.81 ± 425.87 | 102.44 | 3.46 |
|  |  |  |  |  |  |  |  |  |  |  |  |

**Table S3:** Robustness of the method tested with changes in column temperature and flow rate.

| **Parameter** | **Analyte** | **Quality control standard** | **Test condition** | **Analyte added (pg/µl)** | **Analyte detected (pg/µl) (Mean ± SD)** | **n** | **% Accuracy** | **%CV** |
| --- | --- | --- | --- | --- | --- | --- | --- | --- |
|  |  |  |  |  |  |  |  |  |
| Change in column temperature | TMA | LLOQQC | 25°C | 15 | 14.32 ± 0.63 | 6 | 95.48 | 4.45 |
|  |  |  | 30°C | 15 | 15.63 ± 0.02 |  | 104.23 | 0.14 |
|  |  |  | 35°C | 15 | 15.02 ± 0.88 |  | 100.13 | 5.85 |
|  |  | HQC | 25°C | 750 | 755.85 ± 4.58 | 6 | 100.78 | 0.61 |
|  |  |  | 30°C | 750 | 711.62 ± 32.79 |  | 94.88 | 4.61 |
|  |  |  | 35°C | 750 | 785.85 ± 49.76 |  | 104.78 | 6.33 |
|  |  |  |  |  |  |  |  |  |
|  | TMAO | LLOQQC | 25°C | 15 | 15.80 ± 0.62 | 6 | 105.35 | 3.92 |
|  |  |  | 30°C | 15 | 15.21 ± 0.16 |  | 101.43 | 1.08 |
|  |  |  | 35°C | 15 | 14.63 ± 0.97 |  | 97.53 | 6.64 |
|  |  | HQC | 25°C | 750 | 736.35 ± 11.50 | 6 | 98.18 | 1.56 |
|  |  |  | 30°C | 750 | 765.60 ± 55.22 |  | 102.08 | 7.21 |
|  |  |  | 35°C | 750 | 794.93 ± 34.99 |  | 105.99 | 4.40 |
|  |  |  |  |  |  |  |  |  |
|  | Creatinine | LLOQQC | 25°C | 4 | 4.11 ± 0.32 | 6 | 102.72 | 7.70 |
|  |  |  | 30°C | 4 | 3.95 ± 0.19 |  | 98.83 | 4.88 |
|  |  |  | 35°C | 4 | 4.26 ± 0.08 |  | 106.62 | 2.02 |
|  |  | HQC | 25°C | 12000 | 12872.40 ± 690.87 | 6 | 107.27 | 5.37 |
|  |  |  | 30°C | 12000 | 11937.60 ± 301.70 |  | 99.48 | 2.53 |
|  |  |  | 35°C | 12000 | 12452.40 ± 1018.41 |  | 103.77 | 8.18 |
|  |  |  |  |  |  |  |  |  |
| Change in flow rate | TMA | LLOQQC | 180µl/min | 15 | 15.00 ± 0.09 | 6 | 100.02 | 0.58 |
|  |  |  | 200µl/min | 15 | 15.61 ± 0.45 |  | 104.08 | 2.89 |
|  |  |  | 220µl/min | 15 | 15.09 ± 0.85 |  | 100.63 | 5.66 |
|  |  | HQC | 180µl/min | 750 | 728.48 ± 25.17 | 6 | 97.13 | 3.45 |
|  |  |  | 200µl/min | 750 | 758.85 ± 0.93 |  | 101.18 | 0.12 |
|  |  |  | 220µl/min | 750 | 802.43 ± 26.28 |  | 106.99 | 3.28 |
|  |  |  |  |  |  |  |  |  |
|  | TMAO | LLOQQC | 180µl/min | 15 | 14.27 ± 0.70 | 6 | 95.15 | 4.97 |
|  |  |  | 200µl/min | 15 | 15.35 ± 0.17 |  | 102.34 | 1.09 |
|  |  |  | 220µl/min | 15 | 15.79 ± 0.63 |  | 105.24 | 4.00 |
|  |  | HQC | 180µl/min | 750 | 776.40 ± 40.35 | 6 | 103.52 | 5.20 |
|  |  |  | 200µl/min | 750 | 737.10 ± 17.86 |  | 98.28 | 2.42 |
|  |  |  | 220µl/min | 750 | 784.95 ± 55.28 |  | 104.66 | 7.04 |
|  |  |  |  |  |  |  |  |  |
|  | Creatinine | LLOQQC | 180µl/min | 4 | 4.23 ± 0.34 | 6 | 105.86 | 7.97 |
|  |  |  | 200µl/min | 4 | 4.12 ± 0.18 |  | 102.95 | 4.36 |
|  |  |  | 220µl/min | 4 | 3.98 ± 0.07 |  | 99.44 | 1.97 |
|  |  | HQC | 180µl/min | 12000 | 11863.20 ± 573.82 | 6 | 98.86 | 4.84 |
|  |  |  | 200µl/min | 12000 | 12776.40 ± 192.30 |  | 106.47 | 1.51 |
|  |  |  | 220µl/min | 12000 | 12224.40 ± 917.60 |  | 101.87 | 7.51 |
|  |  |  |  |  |  |  |  |  |

**2.8 Matrix factor**

The success of a quantitative cap-LC-MS method for analysis of TMA, TMAO and creatinine in mouse urine lies in the ability of the method to accurately and precisely quantify the analytes in biological samples. The matrix effect was overcome by using ^2^H_9_-TMA, ^2^H_9_-TMAO and acetaminophen as IS and also by dilution of urine. This guarantees high accuracy of cap-LC-MS analysis, by tracking the IS from the initial steps of sample preparation to the cap-LC-MS analysis. The mean CVs of the matrix factor for the three analytes and their corresponding IS were 3.81% and 1.64%, respectively. The CV precision of the normalized matrix factor was 4.69% (Table S4), which indicated that the impact of urine matrix was consistent and limited.

Our method does not require sample pre-treatment, such as solid-phase extraction. Despite the absence of this step there was no evidence of urine metabolites interfering with the quantification of TMA, TMAO or creatinine. The separation step on the C_18_ column and the selectivity of the MS using SIMs obviates the need for sample clean-up.

**Table S4:** Matrix factor for derivatized TMA, TMAO and creatinine and IS derivatized-^2^H_9_-TMA, ^2^H_9_-TMAO and acetaminophen in mouse urine (n=6).

|  |  |  | **Spiked with urine** | |  | **Spiked with water** | |  |  |  |  |
| --- | --- | --- | --- | --- | --- | --- | --- | --- | --- | --- | --- |
| **Analytes** | **QC** |  | **Mean analyte response** | **Mean internal standard response** |  | **Mean analyte response** | **Mean internal standard response** |  | **Matrix Factor (MF) for Analyte** | **Matrix Factor (MF) for internal standard** | **Normalized Matrix Factor (NMF)** |
|  |  |  |  |  |  |  |  |  |  |  |  |
| TMA | LLOQQC |  | 96002 | 387075 |  | 92261 | 394797 |  | 1.041 | 0.980 | 1.061 |
|  | HQC |  | 3434200 | 285180 |  | 3473750 | 287756 |  | 0.989 | 0.991 | 0.998 |
| TMAO | LLOQQC |  | 4673 | 149295 |  | 4640 | 145875 |  | 1.007 | 1.023 | 0.984 |
|  | HQC |  | 90018 | 171652 |  | 88416 | 171106 |  | 1.018 | 1.008 | 1.010 |
| Creatinine | LLOQQC |  | 2431 | 2745660 |  | 2587 | 2732528 |  | 0.940 | 1.005 | 0.935 |
|  | HQC |  | 4233114 | 2633580 |  | 4086858 | 2685690 |  | 1.036 | 0.981 | 1.056 |
| **Mean** |  |  |  |  |  |  |  |  | 0.999 | 1.001 | 1.007 |
| **SD** |  |  |  |  |  |  |  |  | 0.038 | 0.016 | 0.047 |
| **%CV** |  |  |  |  |  |  |  |  | **3.806** | **1.643** | **4.691** |
|  |  |  |  |  |  |  |  |  |  |  |  |

**2.9 Stability**

The stability of TMA, TMAO and creatinine in the stock solutions, urine samples and in the final extract during the processes of sample storage, sample handling, sample preparation and cap-LC-MS analysis was examined. The stabilities of the derivatized TMA, TMAO and creatinine at different conditions are summarized in Table S5.

**Table S5:** Stability of TMA, TMAO, creatinine and their IS under different conditions (n=6).

| **Analyte** | **QC** | **Short-term** | **Freeze-thaw (one cycle)** | **Freeze-thaw (two cycles)** | **Freeze-thaw (three cycles)** | **Autosampler** | **Long-term** |
| --- | --- | --- | --- | --- | --- | --- | --- |
|  |  | **% Accuracy** | **% Accuracy** | **% Accuracy** | **% Accuracy** | **% Accuracy** | **% Accuracy** |
|  |  |  |  |  |  |  |  |
| TMA | LQC | 97.94 | 95.69 | 94.59 | 94.89 | 102.45 | 97.49 |
|  | HQC | 99.54 | 101.25 | 102.52 | 94.25 | 95.47 | 95.53 |
|  |  |  |  |  |  |  |  |
| ^2^H_9_.TMA | LQC | 105.42 | 104.76 | 100.34 | 95.45 | 100.06 | 97.35 |
|  | HQC | 98.61 | 100.22 | 98.92 | 95.45 | 99.80 | 94.93 |
|  |  |  |  |  |  |  |  |
| TMAO | LQC | 95.03 | 100.37 | 96.19 | 98.74 | 98.41 | 93.03 |
|  | HQC | 99.48 | 98.77 | 103.54 | 97.13 | 103.78 | 96.97 |
|  |  |  |  |  |  |  |  |
| ^2^H_9_.TMAO | LQC | 100.71 | 98.27 | 98.01 | 96.78 | 99.13 | 95.18 |
|  | HQC | 98.66 | 102.31 | 105.68 | 96.47 | 106.98 | 97.69 |
|  |  |  |  |  |  |  |  |
| Creatinine | LQC | 99.80 | 98.92 | 99.49 | 97.35 | 103.34 | 94.40 |
|  | HQC | 96.99 | 103.15 | 96.13 | 98.78 | 96.47 | 96.88 |
|  |  |  |  |  |  |  |  |
| Acetaminophen | LQC | 99.96 | 102.23 | 100.98 | 99.24 | 97.67 | 96.21 |
|  | HQC | 100.98 | 97.56 | 98.93 | 97.85 | 104.54 | 98.87 |
|  |  |  |  |  |  |  |  |

LQC and HQC samples were found to be stable when stored at room temperature for 6 h (short-term stability), with accuracies of 95.03% and 105.42%, respectively. Both LQC and HQC samples were stable even after three freeze-thaw cycles of urine. The accuracy of derivatized TMA, TMAO and creatinine measured was 95.69% to 104.76%, at the end of the first cycle, 94.59% to 105.68%, at the end of the second cycle, and 94.25% to 99.24%, by the end of the third cycle. Samples were found to be stable in the autosampler for 24 h at 4°C, with accuracies ranging from 95.47% to 106.98%. The accuracy of quantification of derivatized TMA, TMAO and creatinine after long-term storage (-80°C for 4 weeks) was 93.03% to 98.87%. The stock solutions of derivatized TMA, TMAO and creatinine were stable when stored at room temperature for 12 h and their % accuracy change ranged from -4.23% to 1.67%. When stored at 5°C for 10 days, the % accuracy change of all analyte concentrations in the stock solutions were from -3.54% to 2.02%. Similarly, the change in analyte concentrations in the working solutions ranged from -2.87% to 1.57% and -2.72% to 3.64% during test for the short- and long-term stability, respectively (data not shown).

**References**

[1]United States Pharmacopeia, Validation of Compendial Procedures 1, in: The Official Compendia of Standards USP 37. https://hmc.usp.org/sites/default/files/documents/HMC/GCs-Pdfs/c1225.pdf
